# Non-coding RNAs and their bioengineering applications for neurological diseases

**DOI:** 10.1080/21655979.2021.2003667

**Published:** 2021-12-23

**Authors:** Tuhin Das, Tushar Kanti Das, Anne Khodarkovskaya, Sabyasachi Dash

**Affiliations:** a Quanta Therapeutics, San Francisco, CA, USA; b RayBiotech, Inc, Peachtree Corners, GA, USA; c Department of Neurology, McGovern Medical School, the University of Texas Health Science Center at Houston, Houston, Texas, USA; d Department of Pathology, Weill Cornell Medicine, Medical College of Cornell University, New York, NY, USA; e School of Biotechnology, Kalinga Institute of Industrial Technology, Bhubaneswar, India

**Keywords:** Non-coding RNA, neurological disorder, brain function, bioengineering, non-coding RNA therapeutics, genetic regulation, biomarker

## Abstract

Engineering of cellular biomolecules is an emerging landscape presenting creative therapeutic opportunities. Recently, several strategies such as biomimetic materials, drug-releasing scaffolds, stem cells, and dynamic culture systems have been developed to improve specific biological functions, however, have been confounded with fundamental and technical roadblocks. Rapidly emerging investigations on the bioengineering prospects of mammalian ribonucleic acid (RNA) is expected to result in significant biomedical advances. More specifically, the current trend focuses on devising non-coding (nc) RNAs as therapeutic candidates for complex neurological diseases. Given the pleiotropic and regulatory role, ncRNAs such as microRNAs and long non-coding RNAs are deemed as attractive therapeutic candidates. Currently, the list of non-coding RNAs in mammals is evolving, which presents the plethora of hidden possibilities including their scope in biomedicine. Herein, we critically review on the emerging repertoire of ncRNAs in neurological diseases such as Alzheimer’s disease, Parkinson’s disease, neuroinflammation and drug abuse disorders. Importantly, we present the advances in engineering of ncRNAs to improve their biocompatibility and therapeutic feasibility as well as provide key insights into the applications of bioengineered non-coding RNAs that are investigated for neurological diseases.

## GRAPHICAL ABSTRACT

**Figure:**
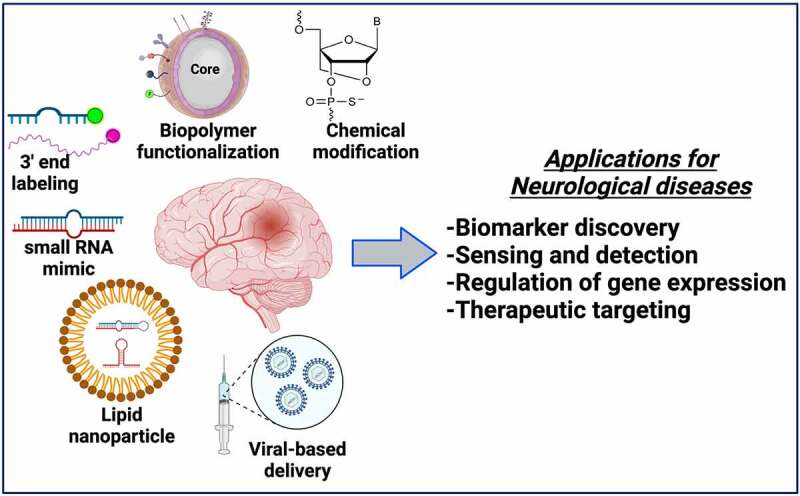

## Introduction

Noncoding RNAs (ncRNAs) are a large cluster of non-protein coding nucleic acid species that have multiple functions in diverse cellular processes. Based on the biological functions and size (i.e., length of nucleotides) ncRNAs are divided into several types such as, ribosomal RNAs (rRNAs), transfer RNAs (tRNAs), small nucleolar RNAs (snoRNAs), small nuclear RNAs (snRNAs), guide RNAs (gRNAs), microRNAs (miRNAs), long non-coding RNAs (lncRNAs) and telomerase RNAs [1–3]. Importantly, regulatory ncRNAs can be classified into microRNAs (miRNAs), small interfering RNAs (siRNAs), long noncoding RNAs (lncRNAs), Piwi-interacting RNAs (piRNAs), promoter-associated RNAs (PARs), and enhancer RNAs (eRNAs) [1–3]. Several ncRNAs perform important roles in a wide range of biological and pathological processes. As such, interest in ncRNA biology has grown throughout biomedicine, biomedical informatics, and clinical sciences. In addition, the field of ncRNA research has witnessed significant progress in recent years due to amalgamation of new-age sequencing ‘omics’ technologies that have contributed to the growing repository of big-data in biomedicine [4,5].

**Figure 1.**
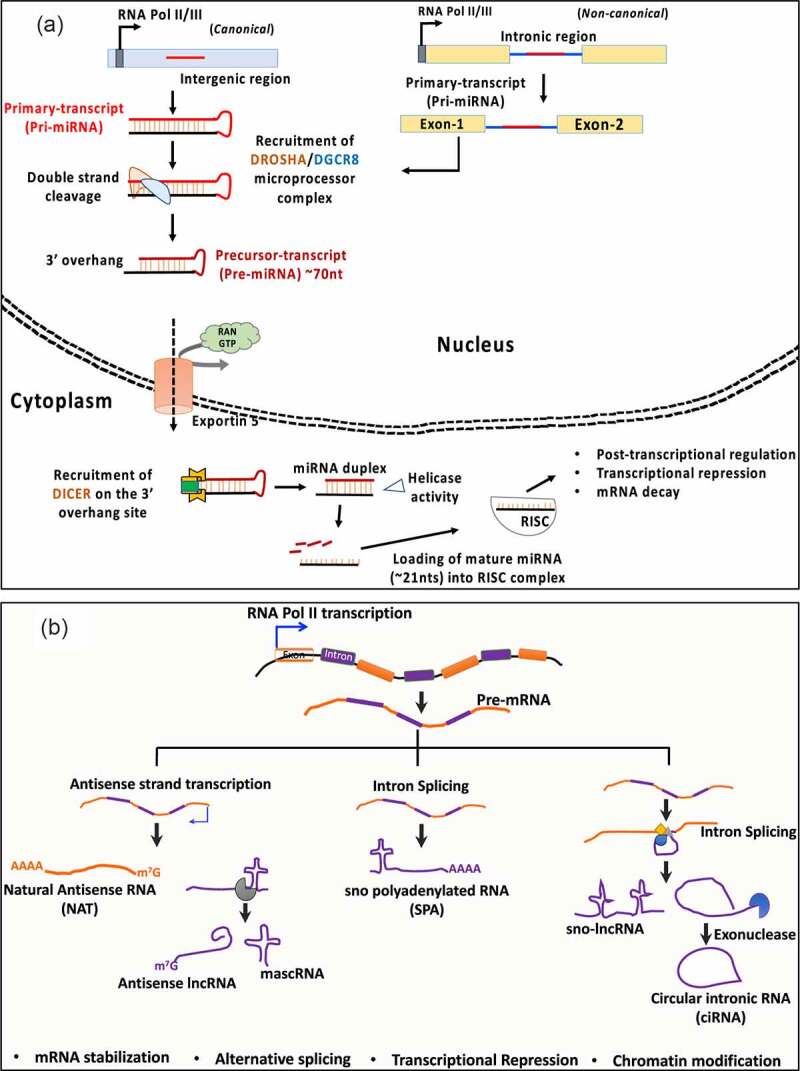
**Cellular fates of miRNA and LncRNA maturation. (a)** microRNAs, transcribed as primary transcripts from intergenic (pri-miRNA) or, intronic regions (mirtrons, non-canonical). DROSHA-DiGeorge syndrome critical region 8 (DGCR8) complex processes the pri-mRNA structures to produce precursor (pre-mRNA) sequence which is exported from the nucleus to the cytosol for further processing by DICER1 and helicase activity to produce a mature 18–22nts long sequence that undergoes ribonucleoprotein (RNP) assembly with the family of Argonaute proteins (AGO1-4), called as RNA-induced silencing complex (RISC) complex. **(b)** Long non-coding RNAs are products of Pol II transcription from intergenic regions. They are presumed to undergo processing events including capping, splicing and polyadenylation, in some cases. *From left –* Natural anti-sense transcripts are transcribed from the opposite(complementary) strands of mRNAs (usually coding in nature). Anti-sense lncRNA and MALAT-1 associated RNA (mascRNA) are produced because of RNAse P processing. U-A-U triple helix at the 3ʹends of mascRNA provides structural stability. sno polyadenylated RNAs (SPA) are 3ʹpolyadenylated and produced from read-through transcripts that are assembled with sno ribonuclear proteins (snoRNPs). In contrast, sno-lncRNAs lack a 5ʹm^7^G cap and 3ʹ poly (A) tail and are produced from excited introns post splicing events. Finally, the circular intronic RNAs (ciRNAs) are also a product of intron excision post splicing events often produced as an outcome of 3ʹexonuclease activity

The dynamic expression and functionality across cell/tissue types has made ncRNAs attractive therapeutic targets. More recently, there have been intensive efforts for establishing siRNAs and miRNAs therapeutics against several diseases including cancer, cardiovascular, genetic disorders as well as to combat viral-induced pathogenesis [6–11]. Currently, there are extensive developments in laboratory investigation approaches to test the therapeutic feasibility of ncRNAs. For instance, the therapeutic feasibility of ncRNAs is being extensively tested in cell and tissue engineering and models of human diseases including cancer, vascular diseases and neurodegeneration by strategies that include the use of antisense oligonucleotides (ASOs), small interfering RNAs (siRNAs), short hairpin RNAs (shRNAs), anti-microRNAs (antimiRs), miRNA mimics, miRNA sponges, circular RNAs (circRNAs) whose details have been reviewed elegantly in previous studies [12–14]. The mainstream approaches for ncRNA-based tissue regeneration therapy include altering endogenous cellular activity using ncRNAs to influence the behavior of resident stem/progenitor cells, cells incorporated into tissue engineered constructs, or modulating the fate of both implanted and endogenous cells with selected ncRNAs [15–18]. MicroRNAs (miRNAs) are 18 ~ 21 nucleotides long, single stranded non-coding RNAs with pleiotropic functions [19,20]. MiRNAs are products of RNA Polymerase II driven transcription which target mature mRNAs in the cytoplasm based on the degree of sequences complementarity (seed, 2–8 nucleotides) between the 5ʹ end of the miRNA and 3ʹregion of the target mRNA (Figure 1). MiRNA-dependent activity is primarily post-transcriptional that results in either mRNA degradation or halting of translation machinery, which in both cases represses target gene expression (Figure 1). On the other hand, lncRNAs are defined as RNA transcripts longer than 200 nucleotides (nts) that are transcribed from intergenic and regions upstream of gene promoters, enhancer-coding regions including the anti-sense strands of protein-coding genes that can exist in both polyadenylated and non-polyadenylated states as illustrated in Figure 1 [1,21–23]. LncRNAs are associated with multiple mechanisms of action, such as chromatin modification, microRNA sequestration by sponging, scaffolding for protein interaction to regulate protein activity, transcriptional regulation by activation or repression *via* transcription factors, mRNA stabilization, splicing modulation, and modulation of translation and degradation of target mRNAs [1,21–23]. A brief description on the maturation of miRNA, lncRNA and other ncRNA species including the circular intronic RNAs (ciRNAs) along with their established cellular functions is illustrated in Figure 1.

It is no doubt that ncRNAs such as miRNAs, lncRNAs, circRNAs are abundant in the CNS. For instance, more than 40% of lncRNAs are expressed in the CNS whereas, several circRNAs and miRNAs are enriched in the neurons and their synaptic junctions. For example, studies have established several ncRNAs including miRNAs (miR-124, miR-125b, miR-21, miR-9), lncRNAs (Gomafu, Neat1) and circular RNAs (CDR1-AS) are highly expressed in the brain in a region or, cell-type-specific manner [24–29]. For instance, miR-124 is abundantly expressed during early stages of embryonic development and neuronal differentiation during adulthood whereas, miR-21 is enriched in the microglia [25]. Similarly, miR-383 is a neuronal enriched miRNA that is abundantly expressed in the brain stem and the cerebellum region [25]. On the other hand, lncRNAs such as Gomafu, MALAT-1 and Neat 1 are implicated in glial specification and synaptic plasticity [30]. Overall, these studies establish the importance of ncRNAs in the development and function of the mammalian brain and present the feasibility of therapeutic targeting of the enriched and abundantly expressed ncRNA species in neurological diseases. The improvement in genomic tools and advances in high power computational algorithms in amalgamation with ‘omics’ technologies has significantly advanced our knowledge on the role of several ncRNAs in phases of brain development to their alterations in diseased states. Neurological diseases or central nervous system (CNS) disorders are broadly defined by the loss of neurons featuring irreversible tissue injury that is manifested in dramatic phenotypic and behavioral changes. Extensive research and current discoveries have established that both miRNAs and lncRNAs are critical determinants of gene expression changes and phenotypic outcomes in neurological diseases. More recently, there is an increasing attention toward understanding the role of circular ncRNA species in healthy and diseased states of the mammalian brain [29,31,32]. Recently there is a major drive in establishing circRNA species as effective stage-specific biomarkers for neuropsychiatric disorders [33–36]. Collectively, the altered expression of ncRNAs such as miRNAs, lncRNAs and circRNAs in circulation (blood, serum, cerebrospinal fluid, CSF) in diseased conditions establishes their importance as biomarkers for disease diagnostics. Therefore, the development of ncRNA based therapeutic approaches could advance the existing repertoire of CNS therapeutics and could further our understanding of ncRNA biology in the healthy and diseased states of the mammalian brain. We acknowledge that the repertoire of ncRNAs in the brain is constantly emerging. However, our understanding of ncRNA regulation and function is primarily based on the evidence gained from miRNAs and lncRNAs. Hence, we critically review the role of ncRNAs (especially miRNAs, lncRNAs) in the major neurological diseases such as Alzheimer’s disease, Parkinson’s disease, along with neuroinflammation and drug abuse disorders that present a global healthcare burden and unmet clinical need. In this review we also discuss on the bioengineering approaches of these ncRNAs especially miRNAs and lncRNAs that are currently underway to improve their therapeutic and diagnostic feasibility for neurological diseases.

## ncRNAs implicated in neurological disorders

ncRNAs confer a high level of regulation by governing gene expression changes at both transcriptional and post-transcriptional levels implicated in the development and function of the CNS. Novel high-throughput molecular and imaging technologies have established their function in neuronal development, differentiation, synapse function as well as in regulation of behavior and cognition. In the recent years an extensive line of evidence has underscored the critical role of ncRNAs in brain development, blood brain barrier formation and regulation, stress response, cellular homeostasis, and neuronal plasticity [37–48]. In addition, loss of function studies have revealed the diseased/pathological outcomes associated with ncRNA function [49–52]. While there are several elegant reviews that can be referred to gain insights into the role of ncRNA species in each of the neurological diseases, in this section we aimed to provide a general overview of the status and role of ncRNAs that are abundantly expressed in the CNS and are dramatically altered in Alzheimer’s disease, Parkinson’s disease and other neurovascular inflammatory conditions.

### Alzheimer’s disease (AD)

Alzheimer’s disease is a complex neurodegenerative disorder and is the predominant cause of elderly dementia [53,54]. The symptoms mark a slow yet progressive memory loss and cognitive decline due to the impairment of region-specific neurons and cellular circuits, which eventually lead to neuronal death/loss. In this regard, several miRNAs and lncRNAs have been identified as key factors that are essential for the regulation of cognitive functions and memory processes in AD [55,56]. For example, in a mouse model of AD (Tg2576 mice), miR-124 was found to be dramatically induced in the hippocampus, which was directly associated with deficits in synaptic communication and memory dysfunction [57]. Studies have shown that expression of several miRNAs was altered in AD pathology, such as miR-30a-5p and miR-128 [58]. In the brains of transgenic animals of AD, a reduction of miR-298 and miR-328 was detected, which was associated to higher β-amyloid precursor protein converting enzyme (BACE1) protein [59]. In related models the level of miR-195 has been reported to negatively correlate with BACE1 protein expression [60]. miR-124 has also been shown to negatively correlate with BACE1 expression in mouse PC12 cells and primary cultured hippocampal neurons exposed to Aβ treatments [61,62]. Overexpression of miR-98 has been shown to induce Aβ production and tau phosphorylation *in vivo*, whereas inhibition of miR-98 exhibits opposing effects [63]. In primary cultures of human brain cells and in brain specimens from AD patients, it has been shown that miRNAs are altered that may have effects on Aβ deposition [64–67].

With regards to lncRNAs in AD pathology, BACE1-AS, a natural antisense transcript (NAT), which is a transcript that is endogenously transcribed from β-amyloid precursor protein cleaving enzyme 1 mRNA sequence has been shown to trigger production and aggregation of Aβ through BACE1 activity, leading to detrimental effects [68]. In a recent study, BC200 RNA was discovered in quantifiable levels specifically in AD patients compared to a gradual decreasing trend seen in the normal age-matched control group [69], and suppressing BC200 inhibited BACE1 expression [70]. It is important to note that the direct role of BACE1 in AD pathology remains unclear. Moreover, the antisense transcript 17A expression has been found to be upregulated *via* the inflammatory signaling leading to increased secretion of Aβ in the cerebral tissue from AD patients [71]. Another natural antisense transcript, NAT-Rad18 that acts by silencing Rad18 mRNA, has reported to be upregulated followed by Aβ-derived neurotoxic apoptosis [72]. Studies have also shown that the complimentary strand of Glial cell-derived neurotrophic factor (GDNF) codes for GDNFOS lncRNA, which shows distinctive expression patterns in postmortem middle temporal gyrus from AD patients compared to age-matched normal group [73].

### Parkinson’s disease

Parkinson’s disease (PD) is considered a multifactorial and aging-related progressive disease, commonly characterized by movement-associated disabilities that depend on the extent of neurodegeneration [74,75]. It is established that motor dysfunction in PD underlies loss of dopaminergic neurons, specifically in midbrain’s substantia nigra [74]. The motor deficits in PD primarily include bradykinesia, rigidity, and tremor [75]. Using high throughput techniques such as RNA sequencing, microarray and microRNA qPCR profiling, studies have reported involvement of several microRNA and lncRNA species in PD pathology [76]. For instance, in the postmortem human brain miR-30b in the substantia nigra and miR-30 c-2 and miR-30d in cingulate gyri were found to be induced in PD patients compared to healthy controls [77,78]. Moreover, induced miR-30b was detected in the exosomes isolated from the cerebrospinal fluid of PD patients [79]. Differential expression levels of miR-30 family members have also been reported in the peripheral blood, plasma and serum of PD patients by several research groups [80–82]. Similarly, miR-29a, miR-29b-1 and miR-29b-2 were upregulated in the anterior cingulate gyri of PD patients [78] Moreover, miR-29b and miR-29 c were downregulated in the PBMCs of PD patients [80] whereas, decreased miR-29a was observed in the blood sample of both drug-treated and untreated PD patients as compared to healthy controls [83]. Based on these observations, it can be suggested that blood serum levels of miR-29a and miR-29 c decrease with the severity of PD pathology [84]. miR-29 has been shown to regulate various processes that are important in PD development, such as apoptosis [85–87], neuronal survival [86], senescence [88,89], motor function [86,90], immune regulation [91–93], and epigenetic modifications [94,95]. Studies have also reported alteration in expression of let-7 family of miRNAs i.e., let-7d-5p, let-7 f-5p and let-7 g in both the prefrontal cortex and blood of PD patients [96]. In the central nervous system, miR-26a was observed to be upregulated in the substantia nigra tissues and in the exosomes isolated from the cerebrospinal fluid of PD patients as compared to healthy controls [77,79], and similar findings have been also reported in the striatal tissues of rodent PD models [97].

On the other hand, recent studies have discovered the involvement of several lncRNA species in Parkinson’s pathology. For instance, NEAT1 levels were significantly upregulated in the peripheral blood of PD patients [98] and has been shown to sponge miR-124 to accelerate Parkinson’s pathology [99]. Homeobox (HOX) transcript antisense RNA (HOTAIR), ~2.2kb nucleotide long non-coding transcript found at the HOXC genetic locus has been reported in PD progression [100]. Studies have revealed a notable decrease in MAPT-AS transcripts encoded from the antisense strand of microtubule-associated protein tau (MAPT) in brain regions of PD-derived tissue [101].

### Neurovascular inflammation

Both miRNAs and lncRNAs have been shown to play crucial roles in the control of brain function [102–104]. Inflammation is one of the important factors that is involved during ischemia-induced brain damage. Pro-inflammatory secretory factors such as IL-1ß, IL-6, TNF-α are robustly induced in response to the damaged blood vessels, tissue injury and cellular apoptosis [105,106]. The combined effect exacerbates brain injury thus making it irreversible. One of the significantly affected signaling pathways is the NF- kß pathway sought to be a prime target for miRNA-mediated regulation [107]. For instance, miR-146a has been shown to inhibit NF-kß signaling by targeting its adaptor proteins, tumor associated necrotic factor 6 (TRAF6) and receptor associated kinase-1 (RAK-1), involved in signal activation [107,108]. miR-146 and miR-9 regulate NF-kß signaling in rodent models of experimental stroke [107,109]. In another study miR-155 was shown to regulate inflammation by promoting TNF- α and IL-1 ß expression *via* toll-like receptor 4 (TLR4) response in transient middle cerebral artery occlusion (tMCAO) model [110]. Stroke-induced ischemic damage induces oxidative stress mediated cell death causing irreversible tissue injury followed by secondary brain damage. Induced reactive oxygen and nitrogen species (ROS and RNS), hydrogen peroxide and free radicals are resultants that subsequently damage the neurovascular unit *via* apoptosis, cellular dysfunction and exacerbated pro-inflammatory signaling [111,112]. Clinically, the additive response of several molecular events underlying stroke pathology determines the infarct volume. Several microRNAs have been accounted to regulate an array of molecular events underlying pathology associated with stroke-induced ischemia [113–116]. For instance, knockdown of miR-23a-3p in the cerebro-ventricular brain region of mice exposed to tMCAO reduced infarct volume in models of ischemia/reperfusion injury [117]. On the other hand, microRNA miR-424 was shown to positively regulate superoxide dismutase activity where knockdown using antagomiRs in the cerebro-ventricular region rescued infarct volume *via* inhibition of cellular apoptosis [118]. In the serum of acute ischemic stroke patients, miR-15a, miR-16, miR-17-5p, miR-125b-5p, miR-125a-5p, miR-143-3P and miR-106b-5p have been detected in elevated levels [119,120]. When miR-106b-5p was inhibited in rat brain post tMCAO, significant recovery was observed in neurological deficit scores and infarct volume [121]. Stroke pathology significantly affects angiogenesis by altering the levels of angiogenic factors (for instance, vascular endothelial growth factor A, VEGF-A) essential for vascular remodeling and functional recovery post-stroke. Over the past few years, several miRNAs have been reported to regulate VEGF-A expression in models of experimental stroke. For instance, miR-140-5p, miR-377, miR-150, miR-107 and miR-210 have been shown to post-transcriptionally regulate VEGF-A mRNA levels by targeting its 3ʹ untranslated region (UTR). Ischemia has been shown to induce angiogenesis *via* the induction of HIF-1alpha dependent VEGF-A/Notch signaling axis and downregulation of miR-153-3p [122].

Other species of small non-coding RNA include small nucleolar RNA (snoRNA), piwi-interacting RNA (piRNA), endo-siRNA, vault RNA, yRNA, tRNA-derived stress-induced RNA, telomere small RNAs, centromere repeat-associated RNA, microRNA-offset RNA, tRNA-derived RNA fragments and splice-site RNAs that are involved in several aspects of development and function [123,124]. Although this diverse family of small non-coding RNAs, microRNAs are the most extensively studied in experimental models of neuroinflammation essentially due to their stability with a well-defined maturation cycle together with the access of current molecular tools and techniques. The depth of knowledge gained from the miRNAs and their function has served as a catalyst toward the development of efficient tools to study the molecular and cellular function of non-coding RNAs.

It is evident that tremendous advances have been made toward understanding the role of noncoding RNAs especially microRNAs. However, the focus is now steadily shifting toward understanding the role of lncRNAs in development and diseases of the mammalian brain. In general, the widespread use of RNA sequencing (RNA-seq) screens has led to the discovery of promising candidates that could pave the way for unveiling the therapeutic potential of ncRNAs. It can also be envisioned that lncRNAs can potentially serve as efficient tools for personalized medicine based on their specific expression patterns in each diseased condition or in a given population. Numerous challenges remain to be resolved before lncRNAs can reach clinical application. The evolving functional repertoire of lncRNAs highlights their involvement in more than one mechanism in diseased states adding layers of complexity to their molecular characteristics. For instance, single nucleotide polymorphisms (SNPs) in ANRIL and H19 have been associated with risks of cancer development and cardiovascular disease [125,126]. Additionally, the low conservation of lncRNAs across evolution of mammalian species is a bottleneck in their discovery and functional validation [127–129]. Based on the knowledge gathered from microRNAs, it is often suggested that the conserved secondary structure of lncRNAs is also of higher importance than its full-length sequence. A key challenge to overcome constitutes the modes of tissue-specific delivery and ensuring the functionality in the targeted cell types of the anti-sense oligos targeting a specific lncRNA. There is a significant lack of in-depth understanding of lncRNA function. Moreover, the translation of lncRNA-based therapy into clinical applications is a long haul that is faced by challenges in understanding the molecular function as well as pharmacological features that include route of delivery, stability of RNA drug in both circulation and cells, duration of treatment, dosage adjustment, and off-target effects. Hence, extensive efforts are needed for a thorough characterization and functional validation of lncRNAs in neuronal and neurovascular pathology, both at the molecular and at the cellular level. A table with the list of miRNAs and lncRNAs well reported in the brain and CNS of patients with neurological disorders are listed in Table 1.

**Table 1.** List of non-coding RNAs (miRNAs, lncRNAs) detected in the brain regions and cerebrospinal fluid of patients suffering from neurological diseases

Alzheimer’s disease		
ncRNA species	Patient brain region	References
mir-29	Hippocampus	[130–133]
miR-15, miR-125, miR-146, miR-222, miR-29	Cerebrospinal fluid	
miR-15, miR-153, miR-455-3p, miR-219	Brain tissue	
miR-46, miR-9, miR-101, miR-106	Cortex, Hippocampus	
miR-125b, miR-107	Cortex tissue	
miR-140-5p	Cerebellum, Hippocampus	
miR-501-3p, miR-93	Serum	
NDM29, 17A	Cerebral Cortex	
GDNFOS, MALAT1	Cerebrospinal fluid	
***Parkinson’s disease***		
miR-30 family	Substantia Nigra	[76,134–137]
miR-29 family	Mesencephalon, Prefrontal cortex	
Let-7 family	Mesencephalon, Prefrontal cortex, Substantia Nigra	
miR-485	Substantia Nigra, Caudate Putamen	
miR-26	Striatum, Substantia Nigra	
miR-200	Midbrain tissue	
miR-151	Prefrontal cortex	
miR-1, miR-28, miR-374	Substantia Nigra	
miR-200a-3p, miR-542-3p, miR-144-5p, miR-151a-3p, let-7 f-5p, miR-27a-3p, miR-125a-5p, miR-423-5p	Cerebrospinal fluid	
AK127687, UCHL1-AS1, and MAPT-AS1	Cerebellum	
HOTAIRM1, lnc-MOK-6:1, and RF01976.1–201	Circulating PBMCs	
lincRNA-p21, Malat1, SNHG1	Brain tissue	
***Schizophrenia***		
GOMAFU	Cortical gray matter, Superior temporal gyrus	[102,138,139]
PINT, GAS5, IFNG-AS1, FAS-AS1	Blood, PBMC in circulation	
***Huntington’s disease***		
HAR1F, HAR1R	Striatum	[133,140,141]
HTTAS_v1	Frontal cortex	
Neat1	Brain tissue	

Disease-specific ncRNAs that have been reported with significantly altered expression in patients are listed in the table have been summarized Alzheimer’s disease, Parkinson’s disease, for Schizophrenia and Huntington’s disease.

It is known that ncRNAs are pleiotropic molecules that exhibit multiple functionalities. With regards to the therapeutic prospects of ncRNAs, it is also important to identify the genetic or molecular targets of ncRNAs that are implicated in diseased states. Therefore, given this pathophysiological importance we have summarized some of the widely reported miRNA and lncRNA species and their experimentally validated genetic targets in respective neurological diseases in Table 2.

**Table 2.** List of non-coding RNAs (miRNAs, lncRNAs) with their respective genetic targets reported in neurological disorders

Alzheimer’s disease		
***ncRNA species***	***Direct and indirect*** ***molecular targets***	***References***
miR-9 family (miR-9-5p)	*GSK-3β, CAMKK2, SIRT1*	[142–144]
miR-146 family (miR-146a)	*ROCK1, CFH, TRAF-6, IL-1, IRAK-1*	[145,146]
miR-125b	*FOXQ1, PTGS2, CDK5, DUSP6, PPP1CA, Bcl-W*	[147,148]
miR-124	*BACE-1, PTPN, NR3C1*	[149–151]
miR-107	*BACE-1, FGF-7*	[152,153]
BACE1-AS	*BACE-1*	[154,155]
51A	*SORL1*	[156]
BDNF-AS	*BDNF, GDNF, EPHB2*	[156]
NAT-Rad18	*RAD18*	[157]
LRP1-AS	*LRP1, HMGB2*	[158]
***Parkinson’s disease***		
miR-30 family	*SNCA, PARK2, LRRK2, ATG5, USP6, NEDD4, USP3*	[78,80,159]
miR-29 family	*DNMT3A, TET2*	[160,161]
Let-7 family	*E2F1*	[162]
miR-181	*p38 MAPK/JNK, PARK2*	[163,164]
miR-26	*PTEN, PINK1, TGF-β/JNK pathway, TAK1, TAB3*	[76]
lincRNA-p21	*sponge for the miR-181 family*	[165]
NaPINK1	*PINK1*	[166]
UCHL1-AS1	*UCH-L1*	[167]
MALAT1	*SNCA*	[135]
HOTAIR	*LRRK2*	[168]
SNHG1	*NLRP3*	[169]
***Schizophrenia***		
AC006129.1	*SOCS3, CASP1*	[170]
Gomafu/MIAT	*QK1, SRSF, SF1, DISC1, ERBB4*	[30,171]
miR-132	*DNMT3A, GATA2, DPYSL3, PTBP2, MMP-9, STAT-4*	[172,173]
miR‐30a miR‐195	*BDNF*	[174]
BDNF-AS	*BDNF*	[175]
miR-212	*MMP-9, MeCP2, STAT4, ZO-1*	[173]
miR-137	*NRG2/3, SYN2/3, ATXN1, ERBB4, GSK3B, FZD3, PTGS2*	[176]
***Huntington’s disease***		
HTTAS_v1	*HTT*	[177]
miR-124	*PGC1, BDNF, SOX9*	[178]
miR-29 c	*P53*	[179,190]
MEG3	*HTT*	[180]
TUG1	*P53*	[181]

Abbreviations of gene names: *GSK-3β (Glycogen synthase kinase-3-beta); CAMKK2 (Calcium/Calmodulin Dependent Protein Kinase Kinase 2); SIRT1 (Sirtuin 1); ROCK1(Rho Associated Coiled-Coil Containing Protein Kinase 1); CFH (complement factor H); TRAF-6 (TNF Receptor Associated Factor 6); IL-1 (Interleukin-1); IRAK-1(Interleukin 1 Receptor Associated Kinase 1); FOXQ1 (Forkhead Box Q1), PTGS2(Prostaglandin-Endoperoxide Synthase 2); CDK5 (Cyclin Dependent Kinase 5); DUSP6 (dual-specificity phosphatase 6); PPP1CA (Protein Phosphatase 1 Catalytic Subunit Alpha); BACE-1 (beta-site amyloid precursor protein cleaving enzyme); PTPN (Protein Tyrosine Phosphatase Non-Receptor); NR3C1(Nuclear Receptor Subfamily 3 Group C Member 1); FGF-7(Fibroblast Growth Factor 7); SORL1 (Sortilin Related Receptor 1); BDNF (Brain-derived neurotrophic factor); GDNF (Glial cell-derived neurotrophic factor); EPHB2 (Ephrin type-B receptor 2); RAD18 (RAD1 Checkpoint DNA Exonuclease 18); LRP1 (LDL Receptor Related Protein 1); HMGB2 (High Mobility Group Box 2); SNCA(Alpha-synuclein); MAPK-JNK(Map kinase- Jun Kinase); PARK2 (Parkin2); LRRK2 (Leucine Rich Repeat Kinase 2); ATG5 (Autophagy Related 5); USP6 (Ubiquitin Specific Peptidase 6); NEDD4 (Neuronal precursor cell-expressed developmentally downregulated 4); USP3(Ubiquitin Specific Peptidase 3); DNMT3A (DNA methyltransferase 3 alpha); TET2 (Ten-Eleven Translocation 2); E2F1 (E2F Transcription Factor 1); PINK1 (PTEN Induced Kinase 1); TAK1(Transforming Growth Factor-Beta-Activated Kinase 1); TAB3(TGF-Beta Activated Kinase 1 (MAP3K7) Binding Protein 3); UCH-L1(ubiquitin carboxyl-terminal esterase L1); NLRP3 (Nod like receptor family pyrin domain containing 3); SOCS3 (Suppressor Of Cytokine Signaling 3); CASP1(Caspase 1); QK1(Quaking Homolog, KH Domain RNA Binding 1); SRSF (Serine And Arginine Rich Splicing Factor); SF1(Splicing factor 1); DISC1 (Disrupted-in-Schizophrenia 1); ERBB4 (Erb-B2 Receptor Tyrosine Kinase 4); GATA2 (GATA-binding factor 2); DPYSL3 (Dihydropyrimidinase Like 3); PTBP2 (Polypyrimidine Tract Binding Protein 2); MMP-9 (Matrix Metallopeptidase 9); STAT-4 (Signal Transducer And Activator Of Transcription 4); MeCP2 (Methyl-CpG Binding Protein 2); ZO-1(Zonula occludens-1); FZD3 (Frizzled 3); NRG2/3 (Neuregulin 2/3); SYN2/3 (Synapsis2/3); ATXN1(ataxin-1); HTT (Huntingtin); SOX9 (SRY-Box Transcription Factor 9); P53 (tumor suppressor protein coding gene 53); PGC1(Peroxisome Proliferator-Activated Receptor Coactivator 1).*

### Other neurological diseases

Dysregulated miRNA biogenesis and activity are directly implicated in the pathogenesis of several complex neurodegenerative and psychiatric diseases. Altered expression of miRNAs such as miR-181b, Let-7 g, miR-26b, miR-30b, miR29b, and miR-106b has been reported in the postmortem brain tissue of schizophrenia patients [182–184]. Fragile X syndrome is a disease characterized by severe mental retardation which is caused by the loss of RNA-binding protein fragile X mental retardation protein (FMRP). Genetic knockdown of FMRP mitigates the effect of miR-125b and miR-132 on dendritic spine morphology [185]. Studies have identified that miR-125b directly targets the NR2A subunit of the N-methyl-D-aspartate receptor (NMDAR), and that glutamate receptor NR2A mRNA which is associated with FMRP, supporting the idea that FMRP functions as a target engagement for miRNA activity [185,186]. Evidence also indicates a strong association between miRNA and Huntington’s disease (HD) pathology given the dysregulation in transcription and processing of microRNAs [187]. In the brains of HD patients, miR-29a, miR-132, and miR-330 have been observed to be expressed in higher levels [188,189]. Significant downregulation of miR-22, miR-128, miR-29 c, miR-138, miR-132, miR-218; and miR-674, miR-344, and miR-222 has also been identified in the mouse models of HD [190]. Several miRNAs such as miR-142-3p, miR-145, miR-146a/b, miR-22, miR-155, miR223/-3p, miR-584, and miR-326 have been also reported to be induced in multiple sclerosis (MS) patients implying their involvement in the pathogenic inflammatory process observed in MS. Dysregulation of various miRNAs targeting the inflammatory activity of various immune cells has also been extensively reported [191–194].

Substance (drug) abuse and addiction are a debilitating disorder characterized by compulsive drug seeking behavior. At the molecular level, it is established that miRNAs play critical roles in synaptic plasticity, neuronal communication and signaling, which are significantly altered in the presence of drugs of abuse [195,196]. For example, multiple studies have demonstrated the involvement of miRNAs in cocaine addiction. Extended exposure to cocaine induces miR-212 concomitant with the increase of total and phosphorylated c-response element binding protein (CREB) in the dorsal striatum of rats, a key region involved in the development of compulsive cocaine use [197]. In a miRNA profiling study of cocaine-induced plasticity genes, it was shown that the miRNAs let-7d and miR-124 are down-regulated, with concomitant induction of miR-181a in the mesolimbic dopaminergic system under chronic cocaine exposure [198,199]. Using next-generation miRNA sequencing method several miRNAs were identified that exhibit cocaine-induced expression changes, such as the miR-8 family members miR-429 and miR-200a/b in the nucleus accumbens and striatal synapses [200]. Recently, the role of poly(ADP-ribose) polymerase-1 (PARP-1) was identified in plasticity, memory, and cocaine addiction [201,202]. Employing *in vivo* models, we reported that PARP-1 was modulated by both miR-125b and miR-124 that were significantly downregulated with cocaine exposure [27,203]. This dose-dependent nature of addiction was implicated in cocaine-induced activation of PARP-1-induced signaling for the maintenance of ATP levels in the dopaminergic neurons [27,203,204]. Specifically, cocaine exposure downregulated both miR-125b and miR-124 in the nucleus accumbens (NAc), however only miR-124 expression was dramatically altered in the hippocampus and ventral tegmental area (VTA) [27,198,203]. Other studies have also found that miR-495, let-7, and miR-212/132 were significantly downregulated in the NAc region upon cocaine treatments that could regulate several canonical reward pathways [205]. Recent studies have also identified lncRNAs that are altered in key reward regions upon cocaine exposure. For instance, lncRNAs TRAF3IP2_AS1 (tumor necrosis factor receptor-associated factor 3-interacting protein 2_antisense 1) and PRKCQ_AS1 (protein kinase C theta antisense 1) are reported to be significantly altered in cocaine abusers [206]. In another study using transcriptome profiling approach, lncRNA Gas5 was identified to be important in inducing cocaine-mediated effects in the mouse nucleus accumbens (NAc) [207]. Using rodent model of methamphetamine abuse, research also reveals dramatic alterations in the lncRNA expression profile in the nucleus accumbens [208].

Collectively, not only these ncRNAs are associated with drug abuse, but they are also associated with several brain diseases suggesting the involvement of novel miRNAs and lncRNAs whose molecular role and functional characterization demands further investigation.

## Bioengineering of ncRNAs for neurological diseases

**Figure 2.**
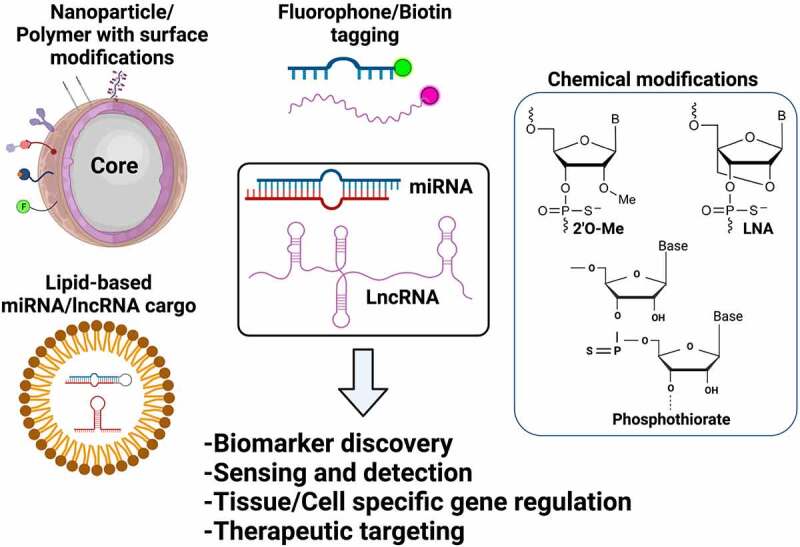
**Bioengineering of ncRNAs for biomedical applications**. Both miRNAs and lncRNAs can be used for several therapeutic and diagnostic applications based on the bioengineering approaches used that include encapsulation of ncRNAs in functionalized nanoparticles, lipid/polymer based nanocarriers, terminal modifications including biotin or fluorophore tagging or chemical modifications by adding functional groups (i.e., 2ʹO-Methyl, P = S bonds) or by bridging the 2ʹoxygen with 4ʹcarbon as represented in the figure

Bioengineering is the application of engineering principles to improve bio-molecular applications to address key challenges in medicine and biology. Bioengineering of ncRNAs can range from nanoparticle-based functionalization to engineering nucleoside chemistry to improve the delivery and pharmacological properties both *in vitro* and *in vivo*. In this regard, bioengineering of ncRNAs is a rapidly evolving field that is anticipated to revolutionize nucleic acid therapeutics for various human diseases including neurological and neurodegenerative disorders. Emerging pre-clinical and clinical evidence has established that miRNAs are secreted in bodily fluids suggestive of effective early-stage biomarkers and therapeutics [209–211]. Therefore, the aim of selectively sensing, enriching, and capturing the changes in miRNA expression profile in a wide range of sample types is critical. Hence, bioengineering of miRNAs *via* magnetic nanoparticle (MNP) or polymer-based functionalization may provide enhanced therapeutic features when compared to traditional approaches [212]. A summary of various bioengineering applications employed for miRNAs and lncRNAs are presented in Figure 2. For instance, using assemblies of nano-(Fe_2_O_3)_ particles in combination with Pt nanoparticles have been used for the detection of miR-21, polydopamine–functionalized (Fe_3_O_4)_ core nanoparticles with carbon dots have been used for sensing of miR-167, and (Fe_3_O_4)_ nanoparticles with carboxyl-modifications have been used in microfluidic devices to quantify miRNA-200a-3p [212]. Moreover, Fe_3_O_4_-Ag (iron oxide-silver) core shell nanoparticles have been utilized for the capture and detection of miRNAs such as miRNA let-7b with an improved sensitivity in the lower femto-Molar (fM) range. Such *proof-of-concept* studies have been performed in a relatively homogeneous system without the interference of blood, cellular factors, complex tissue architecture etc., and thus it should be noted that the number of studies performed in natural body fluids is highly limited. The ability of concentrating circulating miRNAs on the MNPs by surface conjugation or functionalization approaches has resulted in a number of highly efficient miRNA sensing devices [212,213]. Cationic polyurethance (PU) short branch polyethylene imine (PEI) nanocomplexes have been developed as vehicles for miR-145 delivery to inhibit brain tumor by targeting genes such as Sox4 and Oct4, *in vitro* [214]. Similarly, anti-miR-21 nanoparticles co-encapsulating the drug doxycycline have demonstrated enhanced cell apoptosis and reduced tumor growth in brain tumor models [215]. Gold nanoparticles covalently functionalized with miR-182 have been tested to selectively deliver miR-182 to brain tumors that has resulted in reducing tumor size [216]. Similarly, gold nanoparticles loaded with anti-miR-92b in apo-lipoprotein E coated liposomes have been shown to efficiently traverse through the blood brain barrier to reduce tumor size in mouse model of glioblastoma [217]. Another advanced engineering approach includes ‘artificial miRNAs’, where the mature miRNAs sequence is replaced in the primary miRNA transcript for a complementary sequence to target the gene of interest [218,219]. When artificial miRNAs are transfected into mammalian cells, they undergo natural miRNA processing and produce the mature miRNA that directly targets the mRNA due to its sequence complementarity and it is postulated that this approach restricts the off-targets effects of a natural mature miRNA in cells [218,219]. These findings clearly establish that we are beginning to learn about the therapeutic aspects of bioengineering of miRNAs. Additionally, adeno-associated virus 5 (AAV5)-mediated delivery of miRNAs to the brain is currently being investigated as a ‘gene therapy’ in non-human primates in model of Huntington’s disease [220]. The discussed proof-of-concept studies provide a foundation and present the feasibility for investigation of bioengineered miRNAs for neurological diseases such as Alzheimer’s disease, Parkinson’s disease as well as other neuropathologies.

Based on current research trends, the future points toward development of cost-efficient functionalized approaches with improved biocompatibility for early capture, detection and delivery of miRNAs in disease systems [212,213]. Additional engineering of miRNAs includes nucleic acid modifications that improve the stability and target specificity of miRNAs when present in the cellular environment. RNA molecules are unstable due to the presence of a 2′ hydroxyl (OH) group [221]. Other strategies include chemical modifications such as phosphothiorate group, phosphodiester group and lock nucleic acid modifications for miRNA and small RNA-based therapeutics (Figure 2). Therefore, engineering of the nucleosides by incorporation of 2′-*O*-methyl (2′OMe)-modifications improves off-target effects without affecting basal immune response [222,223]. Similarly, 3ʹ cholesterol groups are also used to improve the delivery and bioavailability of small RNAs as cholesterol is a physiological and structural component of animal cell membranes [224].

It must be noted that the diverse functionality of lncRNAs presents multiple ways in which these ncRNAs can be targeted. At the same time these diverse features pose a great deal of challenge in the therapeutic pipeline given the nonspecific events that could arise due to manipulation of a given lncRNA in the cell. However, reports suggest that lncRNAs can be targeted using engineered anti-sense oligos (ASOs) or by manipulating the levels of naturally occurring antisense transcripts (NATs) [13,17,225].

### Therapeutic feasibility of ncRNAs

The use of ncRNAs to treat human diseases is rapidly developing and so are the approaches to engineer ncRNAs for therapeutic and diagnostic compatibility. Several evidence point to the critical role of miRNAs and lncRNAs in disease models ranging from CNS pathologies, drug abuse to viral-induced nervous system disorders [226–230]. As of now, we are beginning to understand the molecular role of lncRNAs whereas, with regards to miRNAs the field is witnessing a transition for small RNA therapeutics [52,231–235]. Several reports have elegantly described various RNA-based therapies that include antisense oligonucleotides (ASOs), short hairpin RNAs (shRNAs), ASO anti-microRNAs (anti-miRs) and small interfering RNAs (siRNAs), that have been developed for liver diseases, muscular disorders, CNS diseases and cancer [12–14]. Currently, approximately 10 RNA-based therapeutics are approved by the FDA and multiple small RNA therapeutic approaches are in stages of clinical investigation for complex CNS and rare genetic disorders [17,225]. Importantly, we and others have discussed on the scope of ncRNA-based therapeutic applications in diseases such as COVID-19-induced neurological disorders, systemic inflammation and cardiovascular diseases [6,230,236–240]. As an example, in 2018, the FDA approved ‘Patisiran’- the first therapy based on administration of RNAi (siRNA-based) for the treatment of rare progressive polyneuropathy caused by hereditary transthyretin-mediated amyloidosis. This drug works by targeting the 3ʹUTR region of transthyretin mRNA [241,242]. Neurological and neurovascular diseases present a global healthcare burden and an unmet clinical need [243–247]. Hence, the observation that miRNAs modulate expression of candidate genes in various diseases has suggested researchers to develop miRNA-based therapeutic strategies [13,231,243,248]. Depending on the data available, a mimic or an antagonist of miRNAs could be explored as therapeutic agent. For example in the case of Alzheimer’s disease, it was shown that the injection of antisense oligonucleotides (ASO) into the CSF of nonhuman primates reduces target RNA (tau) expression in the brain regions analyzed, including the hippocampus [249]. Based on these data, a clinical trial to test a tau ASO in patients with mild AD is currently under investigation by Biogen, IONIS Pharmaceuticals (NCT03186989). Similarly, miRNA silencing is thought to be an attractive therapeutic modality in many other neurodegenerative diseases, including Alzheimer’s disease, Parkinson’s disease and amyotrophic lateral sclerosis (ALS) [250–253]

A recent study focused on identification of unique lncRNAs that could serve as targets for therapeutic agents [254]. Abnormalities of lncRNAs that are involved in many fundamental neuronal cell properties are considered a hallmark of nervous system disorders [24]. Oligonucleotide therapy using an ASO that is single stranded < 40nucleotides may modulate various mechanisms in consequence by binding to complementary targets such as mRNA, miRNA, and NATs [255]. In this context, the drug potential of ASO against lncRNA in Angelman syndrome (AS) was postulated. Patients with genetic defects in AS present with malfunctioning nervous system features, caused by a maternally inherited defect in *UBE3A*, a gene that encodes for E3 ubiquitin ligase [256]. ASO restored *UBE3A* mRNA that was silenced by lncRNA, an antisense transcript of small nucleolar RNA host gene 14 (SNHG14; previously known as UBE3A-ATS) in intact paternal UBE3A *in vivo* [257] and in patients, showing a promising approach to clinical treatment [256]. Moreover, ASOs that target naturally occurring antisense transcripts (NATs) have been shown to induce brain-derived neurotrophic factor (BDNF) *in vivo* to improve neurological outcomes [258]. Importantly, using in vivo models it was discovered that these antisense-NATs targeting BDNF are reported to have higher degree of permeability through the blood brain barrier [258]. In addition, lncRNA MALAT1 was suggested for application as a treatment strategy related to cancer [259,260]. The silencing of MALAT1 by siRNA or ASO has been reported to exhibit positive regulations in *in vivo* models of tumorigenesis by improving features such as tumor cell proliferation, migration, invasion, and apoptosis in various cancer types including lung cancer [261,262], pancreatic cancer [262] and multiple myeloma [259]. LncRNAs have drawn considerable attention in the past decade and currently there is a lack of lncRNA-based or targeting therapeutics in the pre-clinical or clinical domain. However at present most of the attention is diverted on claiming the value of lncRNAs as a diagnostic i.e., stage-specific biomarker. However, it is expected that the field of lncRNAs will witness rapid progress in both basic and clinical sciences.

## Conclusion and outlook

Although we have gained insight into some of the key species of this evolving family of RNA, a vast majority of information is yet to be discovered. The field is currently innovating to overcome the several challenges in developing ncRNA-based therapeutics and diagnostics. It is important to acknowledge that bioengineering of ncRNAs is a relatively young field that needs extensive investigation. The feasibility of chemical modifications, genetic engineering approaches, compatibility of biopolymers, and oligonucleotide biochemistry are some of the key domains that can be advanced by the introduction of interdisciplinary approaches. Considering an example better and improved delivery modalities such as polyethylene glycol, hyaluronic acid or polymers with improved retention, biocompatibility and low degradability and low immunogenicity must be tested using disease relevant *in vivo* models. Another major problem for CNS therapeutics is the site or region-specific delivery of candidate ncRNAs such as, miRNAs to specific diseased sites in the brain. The development of effective miRNA delivery systems is vital as the delivery vehicle must allow miRNAs to cross the blood–brain barrier, which remains a major hurdle in neurodegenerative and neurovascular therapeutics. As miRNAs are easily degradable, the delivery and engineering systems can also be innovated to stabilize and extend the life of the miRNAs. Moreover, host cell endocytosis mechanisms must be exploited to improve the barrier permeability of engineered anti-sense oligos, small RNA mimics or delivery cargos. Similarly, lncRNAs can be investigated for their therapeutic prospects given their direct role in chromatin modifications as well as their direct impact in messenger RNA stabilization and post-transcriptional regulation. Furthermore, it is debated that using ncRNAs as a therapeutic modality may be a double-edged sword due to their multifaceted biological function and pleiotropic properties. Hence, effective methods of bioengineering ncRNAs are postulated as the future to improve our understanding on the therapeutic efficacy and feasibility of ncRNAs for neurological as well as related disorders. Often *in vitro* investigation provides clear cut results however are far from physiological relevance in contrast with *in vivo* approaches that are complex and include species variations and challenges in replication disease etiopathologies. Overall, given the tremendous advances have been made in nucleic acid modifications and delivery systems individually based on specific RNA species, a successful translation will require the integration of interdisciplinary expertise to improve the therapeutic index and feasibility of ncRNAs for neurological and neurodegenerative diseases.

## Highlights

- ncRNAs are critical regulators of gene function and play key roles in brain development and function.
- Bioengineering improves the therapeutic feasibility of ncRNAs for pre-clinical and clinical testing.
- Bioengineering of ncRNAs is key to development of novel diagnostics and therapeutics for neurological disorders.
